# CPA-Cas12a-based lateral flow strip for portable assay of Methicillin-resistant *Staphylococcus aureus* in clinical sample

**DOI:** 10.1186/s12951-023-02002-1

**Published:** 2023-07-22

**Authors:** Jiangling Wu, Yu Huang, Xiaojuan Ding, Lina Kang, Xiaoliang Wang, Dandan Li, Wei Cheng, Gang Liu, Jianjiang Xue, Shijia Ding

**Affiliations:** 1 Department of Clinical Laboratory, Medical Sciences Research Center, University-Town Hospital of Chongqing Medical University, Chongqing, 401331 China; 2 Chongqing Key Laboratory of Multi-scale Manufacturing Technology, Chongqing Institute of Green and Intelligent Technology, Chinese Academy of Sciences, Chongqing, 400714 China; 3 Chongqing School, University of Chinese Academy of Science, Chongqing, 400714 China; 4 Department of Clinical Laboratory, The Second Affiliated Hospital of Chongqing Medical University, Chongqing, 401331 China; 5 The Center for Clinical Molecular Medical Detection, The First Affiliated Hospital of Chongqing Medical University, Chongqing, 400016 China; 6 Department of Critical Care Medicine, University-Town Hospital of Chongqing Medical University, Chongqing, 401331 China; 7 Key Laboratory of Clinical Laboratory Diagnostics (Ministry of Education), college of laboratory medicine, Chongqing Medical University, Chongqing, 400016 China

**Keywords:** Methicillin-resistant *Staphylococcus aureus*, Cross-priming amplification, CRISPR/Cas 12a, Lateral flow strip, Clinical sample, Clinical medication

## Abstract

The rapid and accurate identification of methicillin-resistant *Staphylococcus aureus* at an early antibiotic therapy stage would be benefit to disease diagnosis and antibiotic selection. Herein, we integrated cross-priming amplification (CPA) and CRISPR/Cas 12a (designated as CPA-Cas 12a) systems to establish a sensitive and efficient lateral flow assay to detect methicillin-resistant *Staphylococcus aureus*. This assay relies on the CPA isothermal nucleic acid amplification strategy which can amplify the DNA extracted from *Staphylococcus aureus* and accompanying the indiscriminately trans-cleavage process of Cas 12a/CrRNA duplex after recognizing specific sequence. Taking the advantage of reporter and high turnover Cas 12a activity, a dramatic change in response was achieved to produce a significant increase in the analytical sensitivity. The signal conversion and output were realized using a lateral flow strip to achieve field-deployable detection. Furthermore, this bioassay was accommodated with a microfluidic device to realize automatically portable detection. This proposed assay completed within 30 min with the detection limit of 5 CFU mL^-1^, was verified by testing bacterial suspension and 202 clinical samples. Given the high sensitivity, specificity and efficiency, this colorimetric readout assay through strip could be further promoted to the clinical diagnosis, clinical medication of multidrug-resistant bacteria.

**Supplementary Information:**

The online version contains supplementary material available at 10.1186/s12951-023-02002-1.

## Introduction

Infectious diseases have prominent features in the course of human history. Although there is a significant advance in the control of infectious diseases, especially the eradication of smallpox as the greatest public health achievement to date and on track of the eradication of rinderpest and poliomyelitis, the world is constantly faced with challenges from emerging and reemerging infectious diseases. The discovery of antibiotics is a major step to promote the development of therapy against some infectious agents and mortality has fallen since its advent. However, the excessive and inappropriate use of antibiotics produces the pathogenic bacteria resistance to drug used, and the emergency of resistance pathogenic bacterial strains [1]. Prescription of ineffective antibiotics not only delays effective treatment but also exacerbate antimicrobial resistances in communities [2]. Although hospital-based antibiotic stewardship has benefited the reduction of inappropriate antimicrobial use, hospital length of stay, healthcare cost, rates of resistance etc., there is ongoing challenges exist for antibiotic stewardship programs, such as conundrum of distinguishing bacterial from viral infections, delays in pathogen diagnosis and antibiotic susceptibility due to technological limitations. The advent of precision medicine based on genomics and pharmacogenomics might address some of these challenges facing antibiotic stewardship programs. Rapid identification of altered biology within a patient and guide of therapy choice using the findings has shown potential benefit for improving clinical outcomes [3].

Traditional culture-based bacteriology identification techniques remain the “gold standard” for more than a century in pathogen diagnosis. Although laboratory automation of routine biological cultures has dramatically improved the standardization, throughput, cost-efficiency, the lengthy days required to obtain strain and phenotypic antibiotic susceptibility information remain the bottleneck of physician for efficient therapeutic decision making. Antibiotic therapy is suggested to be carried out as fast as possible since prompt treatment usually resulting in a better outcome. Therefore, accurate identification of etiologic agents is crucial for precise diagnosis, antibiotics selection, infection control and provides clues to underlying sources of infections to guide additional treatment modalities [4, 5]. In addition, antibiotic susceptibility testing (AST) to determine drug-resistant phenotypes is critical to preserve the effective administration of antibiotic [6].

The current assays for pathogen identification and AST profiling could be divided into three classes according to their proximity to the specimen source and required assay time [7]. Class I involves traditional assay techniques that require culture-positive specimens and downstream time-consuming bacteria identification and AST. The techniques in Class II could enhance the analysis speed and accuracy of pathogen identification from culture-positive specimens. The most significant examples are the matrix-assisted laser desorption ionization time-of-flight mass spectrometry (MALDI-TOF-MS) and fluorescence in situ hybridization (FISH) [8, 9]. However, MALDI-TOF-MS relies too much on well-established database to interpret the results, which limits its utilization in resource-limited settings. FISH requires harsh conditions to globally denature genomic DNA for probe hybridization, which may disrupt the integrity of biological structure and increase the likelihood of DNA FISH probes binding to off-target genomic DNA sequences [10]. The feature of the techniques in the Class III is that pathogen identification and/or AST results could be directly analyzed from patient specimens without overnight culture step. The typical examples are the “sample-to-answer” polymerase chain reaction (PCR) assay and lateral-flow assay. PCR method is a good alternative to traditional method in pathogen detection and has been used for clinical testing such as *group B Streptococcus*, *Mycobacterium tuberculosis*, *Mycoplasma*, and *Chlamydia* [11, 12], but the diagnosis process is time-consuming, labor intensive and costly [13]. On the other hand, immunoprecipitation based lateral flow assay is an ideal detection platform for portable diagnostics owing to its outstanding merits of affordability, portability, and user-friendly format [14]. Lateral flow assays have been employed for the detection of various targets ranging from small molecules, biomacromolecules to microorganisms and virus and it is anticipated to boost the growth of the market. Nevertheless, lateral flow assays suffer from relatively poor sensitivity and possibility of generating false-negative results [15].

Strong toxic *staphylococcus aureus* (*S. aureus*) in the clinic is a gram-positive, nonmotile, coagulase-positive coccoid bacterium. The introduction of penicillin in the 1940s significantly reduced the infections caused by *S. aureus* worldwide [16]. However, the discovery of methicillin-resistant *S. aureus* (MRSA) in 1961 that can hydrolyze β-lactam ring and developed resistance to penicillin, has spread all over the world and become one of the notorious pathogens in hospital and community infections. Thermostable endonuclease, an important pathogenic factor of *S. aureus*, is encoded by thermostable endonuclease gene (*Nuc* gene). The presence of *Nuc* in the genome of staphylococcus cells increases the inherent ability of organisms to initiate infection [17]. The mechanism of methicillin resistance in staphylococci is mediated by the *mecA* gene, the product that is the penicillin-binding protein PBP2a with a reduced affinity for β-lactam antibiotics, resulting in resistance to most β-endamine antimicrobial agents [18]. Therefore, we designated two classic genes, *mecA* and *Nuc*, as the two target genes in this strategy.

CRISPR/Cas system as an adaptive immune system of archaea and bacteria has attracted much attention on developing the next-generation diagnosis technology in the field of genome editing as well as nucleic acid-related detection [19]. The versatility of Cas effectors from different families has proven to broadly work for target and cut specific DNA/RNA with high sensitivity and selectivity, including Cas9, Cas12, Cas13 and Cas14 [20, 21]. The easy integration of CRISPR/Cas systems with a range of nucleic acid-based signal amplification techniques and signal probes empowers it with superior properties including high specificity, ultrahigh sensitivity, simple design and easy operation [22]. Moreover, the CRISPR/Cas system can effectively compensate for the risk of false-positive nucleic acid amplification methods in negative sample to improve the specificity and accuracy [23]. However, the signal-readout of the most CRISPR/Cas coupled isothermal amplification methods relies on fluorescent single-stranded DNA, which limits the stability and flexibility in complex samples. Gold nanoparticles have inherent surface plasmon resonance (SPR) property and their advantages in the high extinction coefficient, strong size-dependent SPR, facile surface functionalization and chemical stability have been used in various ways of colorimetric biosensing. The CRISPR/Cas system paired with gold nanoparticles (AuNPs) and lateral flow assays device not only meets the challenges inhered by fluorescent method, but also offers the possibility to develop point-of-care diagnostic tool due to the visible color changes without the requirement of sophisticated instrument [24]. To date, some researchers have dedicated considerable efforts to achieve the portable and quantitative detection of nucleic acids. In 2020, Chen et al. combined a centrifugal microfluidics system and an integrated recombinase-aided amplification (RAA)-CRISPR Cas 12a system for analysis of nucleic acid. This compactable system not only avoids the problems of aerosol contamination but also is more straightforward than the separate reaction [25]. Li et al. proposed the integration of a microfluidic system with CRISPR-based molecular detection for the detection of SARS-CoV-2 RNA in 2022 [26]. The reverse transcription RPA (RT-RPA) amplification techniques was introduced into the microfluidic chip for visual detection read by the naked eye against a lateral flow dipstick. Very recently, Waitkus et al. prepared a localized surface plasmon resonance chip based on the coupling of gold nanoparticles and gold nanomushrooms. This substrate was packaged in a microfluidic cell and coupled with a CRISPR/Cas 13a RNA detection assay, which enabled on-chip PCR for the specific identification of SARS-Cov-2 RNA [27].

Despite the rapid progress in CRISPR-based lateral flow and microfluidic assays, researchers continue to take the initiative in pursuing the improvement of the cleavage efficiency of Cas proteins related to the detection performance. Different techniques for nucleic acid amplification have been incorporated to enhance the detection sensitivity such as rolling circle amplification (RCA), recombinase polymerase amplification (RPA), strand displacement amplification (SDA), primer exchange reaction (PER), catalytic hairpin assembly (CHA). As a complement to PCR, cross-priming amplification (CPA) used one or more pairs of cross-primers is an emerging class of isothermal nucleic acid amplification method. CPA is able to be carried out by a strand displacement DNA polymerase and does not require an initial denaturation step or the addition of a nicking enzyme, which might be advantageous for low-cost assays [28]. At present, this technology is widely used in gene detection, medical disease diagnosis, and so on. Cas12a is capable of protospacer recognition on double-stranded DNA (dsDNA) adjacent motif (PAM) under the guidance of CRISPR RNA (crRNA), and then forms cis-cleavage of the target dsDNA and trans-cleavage of other ssDNA in the system [29]. The unique trans-cleavage activity of Cas 12 only implemented by RuvC domain makes Cas 12 attract abundant attention to the development of novel biosensing techniques. However, to the best of our knowledge, so far, few studies has been explored the potential incorporation of CPA with CRISPR/Cas 12a system combined with lateral flow assays.

Herein, we aim to establish CRISPR/Cas 12a coupled with CPA (CPA-Cas 12a) lateral flow assay for diagnosis of MRSA. The CPA was utilized to amplify the DNA extracted from *S. aureus*, after which beacon was trans-cleaved by Cas12a-CrRNA duplex on the basis of specific sequence recognition. The reporter and high turnover of Cas 12a would be used to produce dramatic signal response specific to each unique gene targets. Lateral flow strips were designed to accommodate readout CPA-Cas 12a system. Furthermore, this lateral flow strip was incorporated to a microfluidic device to realize portable detection. Bacterial suspension and clinical samples were selected to prove the reliability of the proposed method. With great accurate and efficiency, CPA-Cas 12a platform made a promising alternative to molecular diagnosis of bacteria and may open possibilities for high-performance isothermal amplification technology.

## Experimental

### Materials and reagents

In this experiment, the silicon magnetic beads used for nucleic acid extraction were purchased from Daan Gene (Guangzhou, China). Guanidine hydrochloride (Gu-HCl) was supplied by Macklin Biochemical Technology Company (Shanghai, China). MRSA PCR kit was purchased from Biorab (Beijing, China). Lateral flow strips with the capability of detection 1 or 2 amplicons simultaneously, were purchased from HUNTARRAY Inc. (Jiangsu, China). Cas 12a protein was purchased from New England Biolabs (Beijing, China). Lysozyme, betaine, dNTP, BstDNA 2.0 polymerase and RNase inhibitors were purchased from Sangon (Shanghai, China). Polymethyl methacrylate (PMMA) microfluidic devices (including optic-grade polycarbonate film) was manufactured by Zhongxinhengchuang Electronic Technology Co., Ltd. (Beijing, China). All oligonucleotides (sequences in Table S1) were synthesized by Sangon and purified by high performance liquid chromatography. They were dissolved and diluted to 10 µM in TE buffer (10 mM, Tris-HCl, 1 mM EDTA, pH 8.0), stored at -20 ℃ for further use. All other reagents were of analytical reagent grade. All solutions were prepared by ultrapure water with resistivity of 18.2 MΩ·cm.

### Instruments, software, and statistical analysis

The Ø 5 mm circular Ru-iron-boron magnet (Ningbo, Kerong Magnetics Inc., China) was employed in the microfluidic device. The micro ceramic heating apparatus (XH-RP1010, Huilide, Shenzhen, China) was powered by a direct current power supplier (JP1560D DC, Wuxi Annas Power Supply Inc., China). The surface temperature of ceramic heating apparatus was monitored by an infrared thermal imager (H13, Hikmicro, Hangzhou, China). Teflon tubes, connectors, and other consumables were provided by Zhongxinhengchuang Electronic Technology Co., Ltd. (Beijing, China). Fluorescence spectra and gel electrophoresis images were performed with integrated steady-state transient fluorescence spectrometer (Edinburgh FS5, England) and chemiluminescence imaging system (Vilber, Fusion Solo S. EDGE, France) respectively. The strip images were collected by a camera embedded in the iPhone 13 smartphone and their uniform quantitative analysis was performed by Image J. SPSS 20.0 was used for statistical analysis.

### Bacterial preparation

*S. aureus* subspecies (MRSA, ATCC 43,300, methicillin-resistant, second-generation species) was purchased from BeNa Culture Collection (Beijing, China). *S. aureus* (ATCC 29,213), *Streptococcus pneumoniae* (*S. pneumoniae*, ATCC 49,619), *Escherichia coli* (*E. Coli*, ATCC 25,922), *Pseudomonas aeruginosa* (*P. aeruginosa*, ATCC 27,853) and *Candida albicans* (ATCC 10,231) were obtained from quality-control strains in microbiology laboratory of University-Town Hospital of Chongqing Medical University. *Enterococcus faecalis* (*E. faecalis*) *Enterobacter cloacae* *E. cloacae* *Klebsiella oxytoca* were obtained from clinical wild strain in microbiology laboratory. All strains including the resistance of MRSA to penicillin and cephalosporins were confirmed by the phoenix 100 bacterial identification instrument (BD, USA). All strains preserved in the original bacterial suspension (72 mM K_2_HPO_4_, 3.8 mM sodium citrate, 0.7 mM MgSO_4_, 88% glycerol) were cultured on Blood, MacConkey or Sabouraud agar plate medium respectively in an incubator containing 5% CO_2_ at 37 ℃ for 12–24 h. Bacterial suspensions with concentration of 0.5 MCF determined by a scattered light turbid meter according to the optical density measured at the wavelength of 600 nm in PBS were prepared for nucleic acid extraction or other experiments.

### CPA-Cas 12a detection

The *S. aureus* DNA was extracted before analyzed with the CPA-Cas 12a detection system. The CPA amplification system was composed of eight primers (0.4 µM *mecA*-CF, *mecA*-CB, *nuc*-CF, and *nuc*-CB; 0.8 µM *mecA*-DF, *mecA*-DB, *nuc*-DF and *nuc*-DB), 0.5 mM dNTP, 2 µL 10 × Bst buffer, 6 U BstDNA polymerase large fragment, 4 mM MgSO_4_, 0.5 M betaine, 1 µL bacterial DNA extraction solution. The mixture was incubated in the centrifuge tube at 63 ℃ for 30 min. Then, CRISPR/Cas 12a amplification procedure was performed with CPA. A total of 100 nM CrRNA was preincubated with 75 nM CRISPR/Cas 12a protein in 1 × NE Buffer at 37℃ for 10 min. After the CRISPR/Cas 12/CrRNA complex was successfully formed, the CPA-Cas 12a trans-cleavage assay was performed in a 50 µL mixture containing 25 µL of 2 × NE Buffer, 1.25 µL Beacon (Fam-TTATTATTATT-Biotin, 10 µM), 1 µL CPA amplification products, 9 µL CRISPR Cas 12a/CrRNA protein mixture, 13.75 µL deionized water containing RNA enzyme inhibitor (1 U µL^− 1^) reaction system. The assay was carried out at 37℃ for 30 min, followed by lateral flow strip detection. In the optimization studies, three replicate tests were carried out to the same sample and the average value was calculated.

### Design of lateral flow strips and microfluidic device

**Fig. 1:**
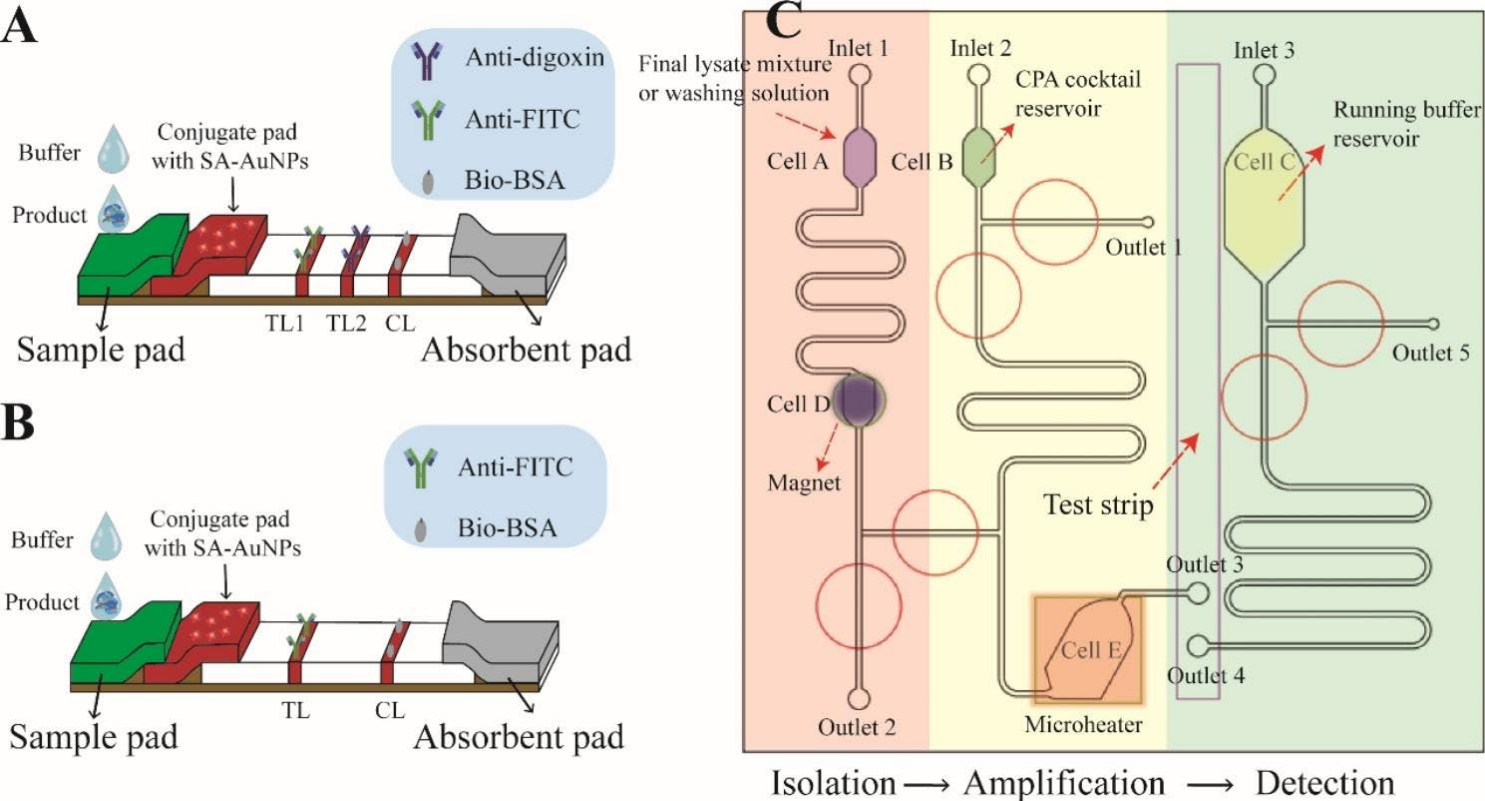
Schematic diagram of two kinds of test strip and microfluidic device for detection. **(A)** Single amplification strip mediated by CPA **(B)** Double amplification strip mediated by CPA-Cas 12a. **(C)** Schematic illustration of microfluidic device

To compare the advantages of CPA and CPA-Cas 12a based amplification strategies, we designed two kinds of test strips for DNA detection as shown in Fig. 1. The first kind of strip can detect two genes simultaneously by CPA (Fig. 1A). Control line (CL), test line 1 (TL1) and test line 2 (TL2) were coated with biotinylated BSA protein, anti-FITC antibody and anti-digoxin antibody respectively. We introduced FITC and digoxin labeled primer DF into the CPA amplification system with two genes respectively, while added biotin labeled primer DB (Fig. S1). Therefore, digoxin-Biotin presented in the amplification products of *mecA*, while FITC-Biotin presented simultaneously in the amplification products of *Nuc*. After SA-AuNPs capturing biotin, the *mecA* products can bind to anti-digoxin on TL2, and the *Nuc* products can bind to anti-FITC on TL1, achieving simultaneous detection of these two genes. The second kind of strip using CPA-Cas 12a was able to detect a single gene (Fig. 1B). The CL and TL were coated with anti-FITC antibody and biotinylated BSA protein respectively. The CPA amplification product of the target gene could be recognized by the Cas12a system. Cleaving the single stranded DNA with two ends modified with biotin and FITC resulted in the loss of cross-linking between biotin and FITC. AuNPs cannot be crosslinked through AuNPs-SA-biotin-DNA-FITC-anti-FITC bridge. As a result, TL did not show any color change. On the contrary, the color of red appeared at TL without target gene.

The microdevice shown in Fig. 1C consisted of three functional regions, which were DNA separation zone, isothermal amplification zone and detection zone respectively. The DNA extraction reagents, CPA cocktail, and running buffer (TE buffer, 10 mM Tris, 1 mM EDTA, pH 8.0) were stored in the external injection tube, liquid reservoir B and liquid reservoir C respectively prior to test. A magnet was arranged under the storage cell D to control magnetic beads. A microheater was arranged under storage cell E to provide a suitable temperature for cross-priming amplification. The liquid flows direction was controlled by six pressure flow switches (red circle). Magnetic beads were injected into storage cell D in order to extract DNA prior to its detection. The presence of chaotropic salts could strengthen the hydrogen bonding interaction between the surface of magnetic beads and DNA. Then, extracted DNA was washed off from the surface of magnetic beads and flowed into the isothermal amplification zone cell E. This elution solution was then mixed with CPA cocktail here, and reaction was allowed to continue for 60 min at 63 °C. Finally, the product was dropped on the lateral flow strip via the connecting channels patterned on the second layer for qualitatively further analysis.

### Patients

166 patients with infection identified at University-Town Hospital of Chongqing Medical University from May to October 2022 were enrolled in this clinical study. The criteria used to select patients include: (1) Clinically diagnosed with infectious diseases; (2) Able to obtain secretions from the infected tissues; (3) Treatment records, imaging, debridement, antibiotic use, and other clinical data were available. 202 pieces of secretions and clinical pathological data were collected from these infected patients for analysis. This study was approved by the Ethics Committee of the University-Town Hospital of Chongqing Medical University, and all participating patients had signed the informed consents.

## Results and discussion

### Principle of CPA-Cas 12a lateral flow assay strategy

**Fig. 2:**
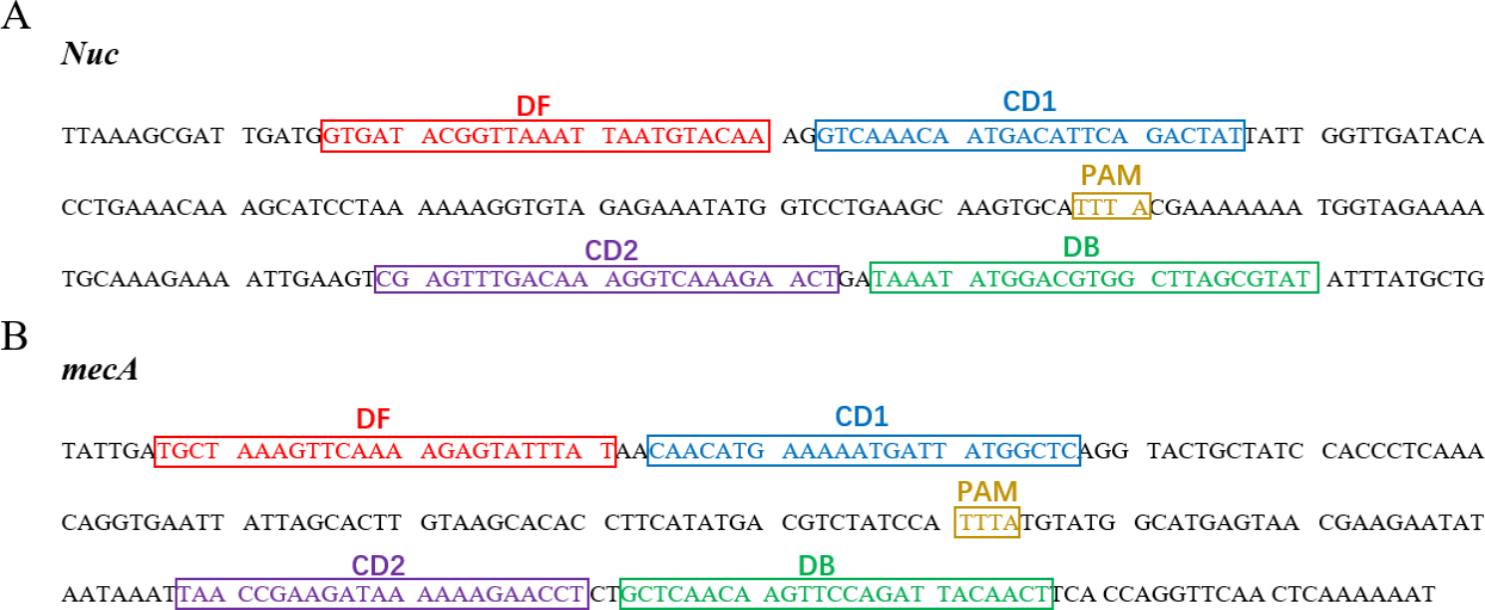
Schematic diagram of primer design for CPA and CPA-Cas 12a assay. **(A)** Primers of *nuc* and PAM, **(B)** Primers of *mecA* and PAM.

CPA and CPA-Cas 12a sensing strategies were proposed for detection of *nuc* and *mecA* genes in MRSA respectively. According to the sequence of the target gene, we designed two PAM sites and four pairs of primer according to the principles of double-crossing CPA and trans-cleavage of CRISPR/Cas 12a. The details of PAM sites and primers are shown in Fig. 2 and Table S1 respectively.

**Scheme 1:**
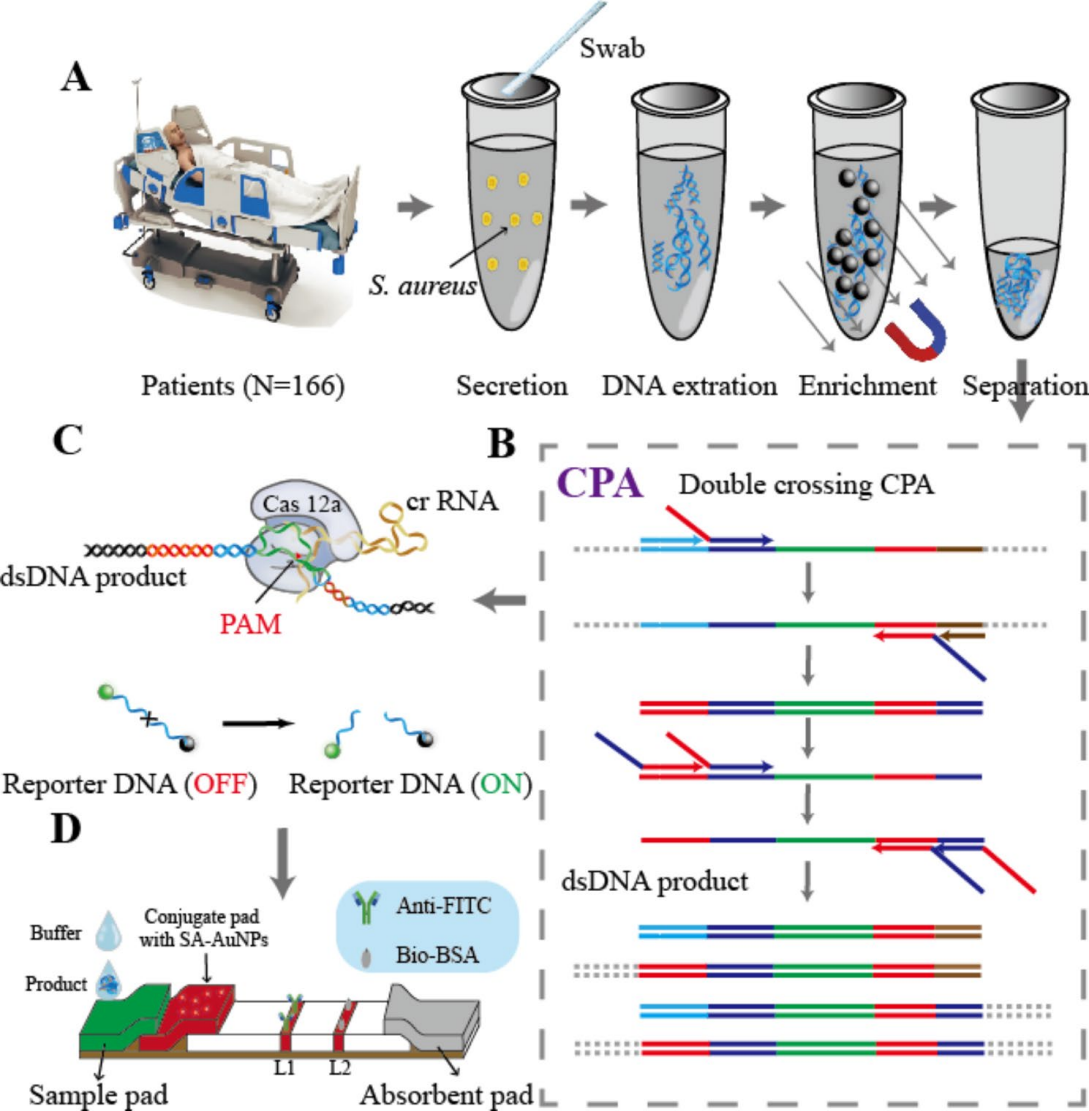
Schematic illustration of CPA-Cas 12a mediated lateral flow assay for MRSA. **(A)** Sample preparation from patient. **(B)** Design of CPA system. **(C)** CRISPR/Cas12a activity. **(D)** Visualization principle of lateral flow strips

The outline of double signal amplification CPA-Cas 12a assay for MRSA in the secretions of infected patients is described in Scheme 1. As shown in Scheme 1 A, 500 µL lysate solution containing 20 mg mL^− 1^ lysozyme and proteinase K was added to enrolled patients’ secretions for 30–60 min reaction at room temperature. Next, 500 µL mixture (50 mg mL^− 1^ silicified magnetic beads, 8 M GuHCl) was introduced to as-prepared lysate solution. The reaction was incubated at room temperature for 20 min and stirred slightly at every 5 min. Subsequently, magnetic beads were separated and cleaned twice with cleaning solution (25 mM NaHCO_3_, 1% casein, 0.4 mM EDTA, 0.2% α-cyclodextrin, 0.2% Triton-100, 0.4 M urea, 0.1% sodium azide, pH 9.6). Finally, 200 µL eluent (35% absolute ethanol) was used to eluate the nucleic acid absorbed on the surface of magnetic beads, which was the analyte in the following study.

The process of CPA is shown in Scheme 1B. Efficient isothermal amplification of target sequence was able to be achieved by using two pairs of cross-primers. The produced four kinds of dsDNA involving target sequence could be recognized by CRISPR/Cas12 system under the guidance of CrRNA. The repeat sequence and programmable target-specific sequence designed in CrRNA promote dsDNA to form an R loop. In presence of Cas 12a, formed Cas12a-CrRNA duplex can specifically recognize and cis-cut target dsDNA (Scheme 1 C). After the reaction, nonspecific ssDNA can be trans-cut by duplex. In this study, a large amount of dsDNA generated by CPA was recognized by Cas 12a protein and CrRNA, resulting in dissociation of reporter DNA. Therefore, the established lateral flow strip biosensor could convert the disintegration of reporter DNA into visual detection (Scheme 1D).

### Verification of CPA integrated CRISPR/Cas system

As shown in Fig. S1A, we designed two pairs of primers labeled with FITC and digoxigenin to evaluate the feasibility of amplification reaction of double-crossing CPA system. After reaction, the products were examined by agarose electrophoresis as shown in Fig. S1B. A large amount of dsDNA products with different chemical base could be seen in lane 5 and 7 at the expense of primers depletion, which demonstrated the successful development of this CPA isothermal amplification method.

**Fig. 3:**
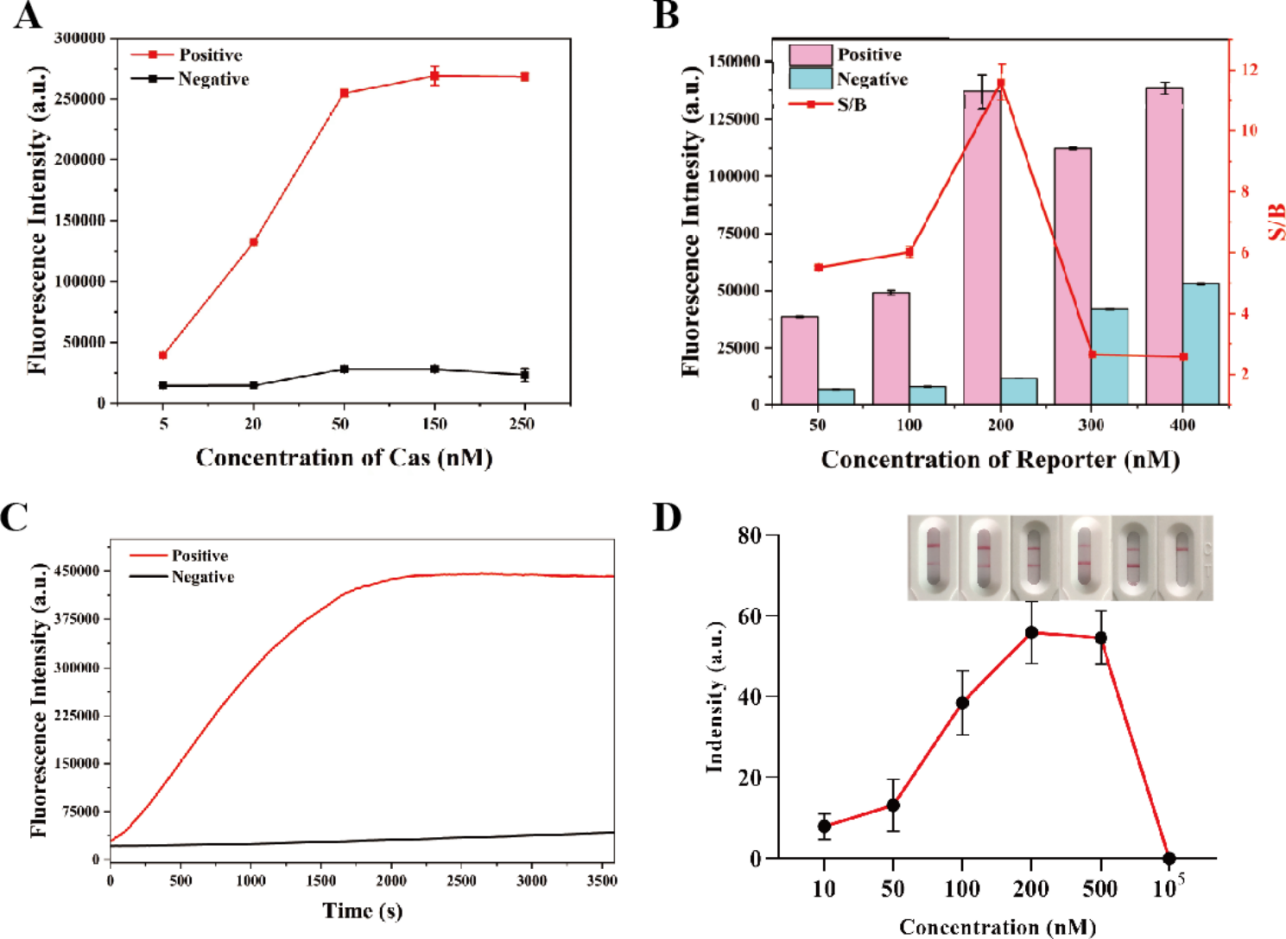
Optimizations of concentrations of **(A)** CrRNA and Cas 12a, **(B)** beacon, **(C)** reaction time of double-crossing CPA-Cas 12a assay and (D) beacon on lateral flow strip

On the other hand, a total of 100 nM CrRNA was preincubated with 75 nM CRISPR/Cas 12a protein in 1 × NE Buffer a 37 ℃ for 10 min. After the successful formation of CRISPR/Cas 12/CrRNA complex, the CPA-Cas 12a trans-cleavage activity was performed in a 100 µL mixture containing 25 µL of 2 × NE Buffer, 1.25 µL DNA reporter molecule (FITC-ATTAGCACTTGTAAGCACACCTTCA, 10 µM), 1 µL aforementioned CPA amplification products, 9 µL CRISPR Cas 12a/CrRNA protein mixture, 13.75 µL deionized water containing RNA enzyme inhibitor (1 U µL^− 1^) reaction system. The reaction was carried out at 37℃ for 30 min and the product was characterized by agarose electrophoresis. As shown in Fig. S2, lane 1, lane 2, lane 3, lane 4, lane 5, lane 6 and lane 7 were the bands of marker, CrRNA, CrRNA with Cas12a, CPA product, DNA reporter, CPA with CRISPR/Cas 12a product and Cas12a-CrRNA duplex without CPA products respectively. By comparing the bands in lanes 5, 6 and 7, DNA reporter was able to be trans-cleavage by Cas12a-CrRNA duplex to form fragment in the presence of CPA product containing target dsDNA, resulting in disappearance of bands marked as red arrow. In addition, in order to demonstrate that the CPA products could initiate ssDNA trans-cleavage activity of Cas12a-CrRNA duplex, a fluorophore and quenched labeled ssDNA reporter denoted as Beacon_2_ with 5 bases (Table S1) of approximately 1.7 nm in distance, was designed and added in the above-mentioned 100 µL mixture without DNA reporter molecule. As shown in Fig. 3C, the fluorescence was recovered when only CPA products and Cas12a-CrRNA duplex simultaneously presented to start the trans-cleavage activity, which further demonstrates the excellent specificity of proposed CPA-Cas 12a strategy.

### Optimization of the assay conditions

To achieve the best signal output performance, various factors including concentrations of CRISPR/Cas 12/CrRNA complex and beacon in solution, reaction time, and the concentration of beacon on strip were optimized through repeated assays.

We firstly optimized the concentration of CRISPR/Cas 12/CrRNA complex according to our previous work [30]. As shown in Fig. 3A, the magnitude of fluorescence increased from 5 nM, and started to stabilize at 50 nM. After 50 nM the fluorescence showed no obvious change. In contrast, the negative control (without bacterial DNA extraction solution) signal did not change significantly with the increase in the concentrations of CrRNA and Cas 12a, further demonstrating the high specificity of this assay. Therefore, a concentration of 50 nM was chosen for CRISPR/Cas 12/CrRNA complex. To ensure high sensitivity and efficiency, we varied the concentration of beacon from 50 to 400 nM. Figure 3B shows the concentration of beacon dependence of fluorescence response in the presence and absence of target respectively. According to the signal/background, which was to quantify the sensitivity of assays, beacon with 200 nM afforded the highest efficiency. Thereby, 200 nM beacon which could provide the signal-to-background ratio (S/B) of 11 was selected in our experiment. Furthermore, the time-dependent change of fluorescence signal was recorded after target was introduced. Figure 3 C showed that after the addition of target, the fluorescence kept increasing gradually in early time and its fluorescence intensity reached the maximum when the incubation time was close to 1800 s, while the negative control without bacterial DNA extraction solution hardly affected the fluorescence intensity in the duration of 3500 s. In addition, the concentration of beacon on the strip exhibited the same optimal concentration of 200 nM in homogeneous phase as that selected in solution phase as shown in Fig. 3D. It is worth mentioning that when the beacon concentration increased to be 10 µM, the signal output showed a false-negative, which may be related to the hook effect. Therefore, this CPA via Cas 12a mediated fluorescent signal and its coupled lateral flow assay has the same disadvantage of high-concentration inhibition. In order to circumvent this drawback, we proposed this CPA-CrRNA based reverse signal output mode to switch the concentration of DNA reporter molecules to visual signal, where the target dsDNA and the signal collected from test line had inverse proportional relationship.

In short, the following experimental conditions were found to give best results: (a) 50 nM of CRISPR/Cas 12/CrRNA complex; (b) 200 nM of beacon in both homogeneous phase and lateral flow strip; (c) reaction time of 30 min.

### Analytical performance of CPA-Cas 12a lateral flow assay

**Fig. 4:**
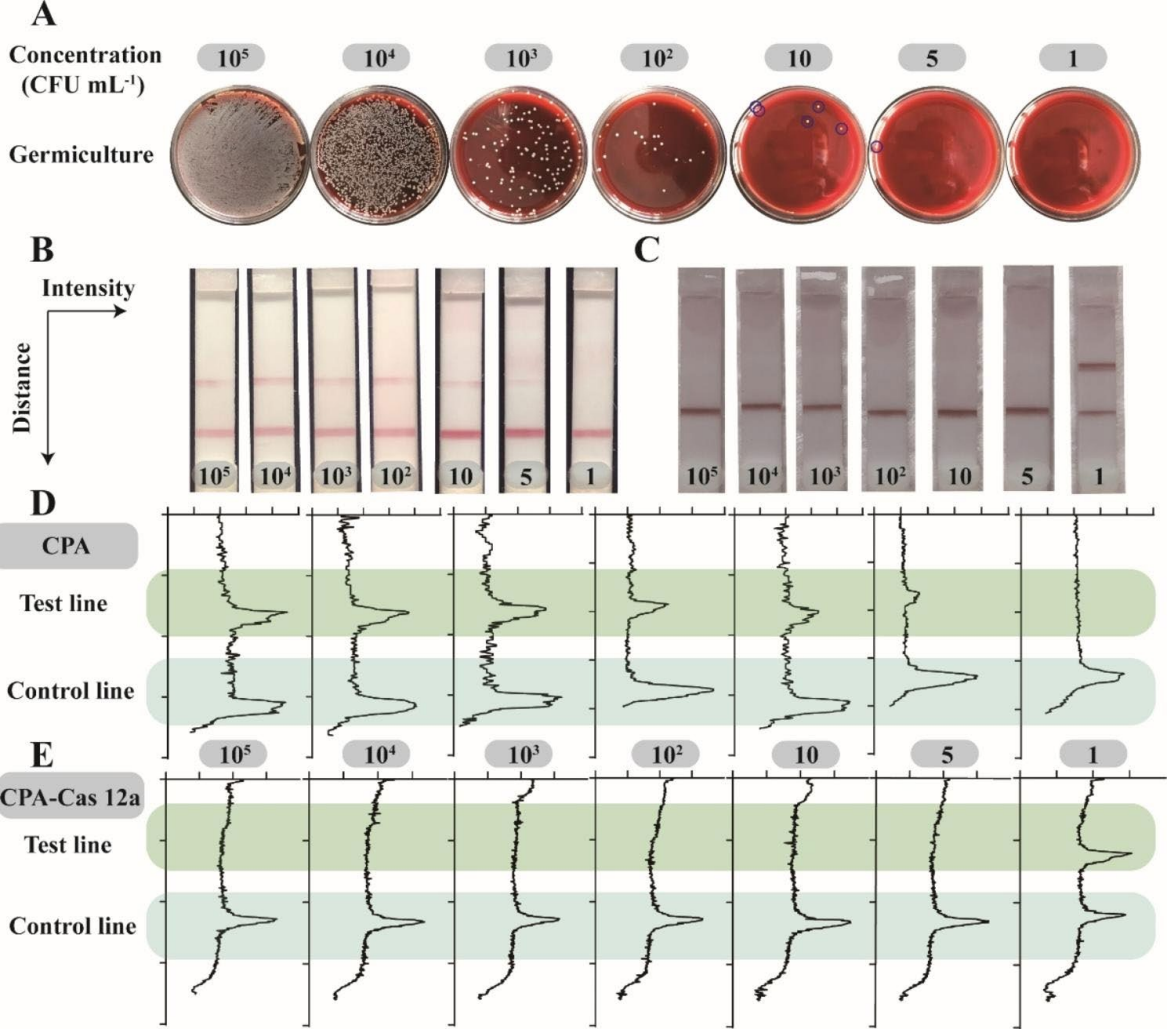
Verification of CPA-Cas12a mediated lateral flow assay (*nuc*). **(A)***S. aureus* absolute colony culture counts of *S. aureus* at different concentrations. **(B)** Results of CPA mediated lateral flow strips for detecting bacterial suspensions with different concentrations. **(C)** Results of CPA-Cas 12a mediated lateral flow strips for detection of bacterial suspensions with different concentrations. **(D)** Densitograms of line in CPA mediated lateral flow strips; **(E)** Densitograms of line in CPA-Cas 12a mediated lateral flow strips

Under optimal experimental conditions, the analytical performance of this lateral flow strip assay was evaluated by detecting different concentrations of *S. aureus*, which was prepared by diluting 0.5 MCF (~ 0.5 × 10^8^ CFU mL^− 1^) of *S. aureus* bacterium suspension with different volume of PBS. As shown in Fig. 4A, germ culture was used to prepare every diluted bacterium suspension and their bacterium colony was counted by blood plate. The concentration of 500 µL bacterial suspension was adjusted to 1, 10, 10^2^, 10^3^, 10^4^ and 10^5^ CFU mL^− 1^ to extract DNA and initiate CPA-Cas 12a reaction. The lateral flow strip detection was executed as described in the last section. All experiments were repeated in three times. Figure 4B and C exhibit the performance comparison of CPA based single-amplification assay and double amplification CPA-Cas 12a based assay respectively. *S. aureus* spiked in PBS could be successfully identified on both kinds of lateral flow strips by naked eyes within 3 h. The intensity of test and control lines of lateral flow strips representing as densitograms was analyzed by Image J after homogenizing as show in Fig. 4D and E respectively.

**Fig. 5:**
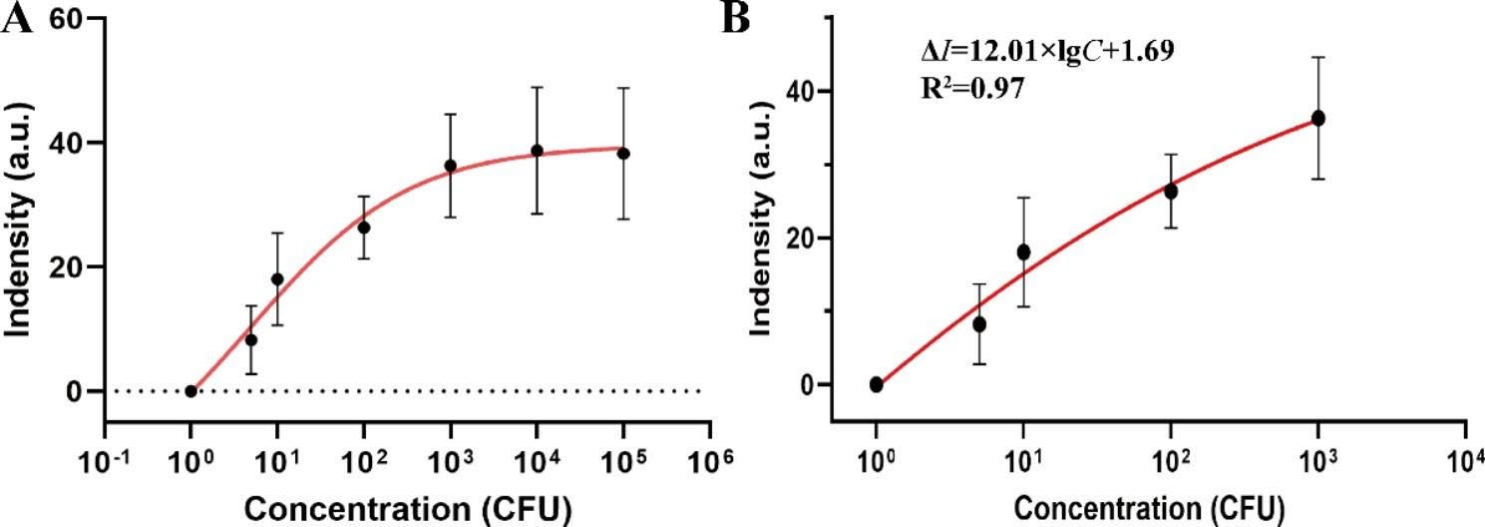
Sensitivity evaluation of the assays. **(A)** Color intensity of T line in CPA mediated lateral flow strip for different concentrations of bacterial suspension. **(B)** Linear curve fitting of color intensity with bacterial concentrations ranging from 1 to 10^3^ CFU mL^− 1^

The signal intensity of CPA based assay increased continuously with the increase of *S. aureus* concentration (Fig. 5A). As shown in Fig. 5B, a good linear correlation between the value of signal and the *S. aureus* concentration between 1 and 10^3^ CFU mL^− 1^ was obtained with regression equation of Δ*I* = 12.01×lg*C* + 1.69 (Δ*I* represents the intensity of test line, and *C* represents the bacterial concentration) with a correlation coefficient (R^2^) of 0.97, and the detection limit was calculated to be 2.26 CFU mL^− 1^, evaluated by the definition of the rule of 3σ/k (σ represents the standard deviation and k represents the intercept of the fitting curve) [31]. But given the potential limitations of visual inspection and actual detection capability, we estimated the LOD of our CPA based biosensor by naked eye was 5 CFU mL^− 1^.

Since the gene of *S. aureus* could be amplified with CPA, the resultant CPA amplicon could activate the ssDNA trans-cleavage activity of Cas12a-CrRNA duplex and produced the beacon from breakage of bridge connection. Thus, the CPA product originated from *S. aureus* could be visually analyzed by observing the color intensity in the test line coated with AuNPs-streptavidin conjugates as well as anti-FITC by hapten-anti-hapten interaction. To quantify the magnitude of color intensity in the test line on the lateral flow strips, densitograms of its corresponding pixel were employed to calculate its optical density as shown in Fig. 4E. The optical density of test line exhibited a nonlinear relationship with the quantity of bacterial, which no signal was detectable in the presence of *S. aureus* at the concentration larger than 1 CFU mL^− 1^. This is attributed to this ultra-sensitive double-amplification CPA-Cas 12a sensing strategy. Furthermore, this ultra-sensitive “0” or “1” binary output mode could significantly increase true positive rate, reduce false negative rate, and show an excellent advantage for semi-quantitative analysis by visual inspection (LOD = 5 CFU mL^− 1^). The analytical performance of this method was compared with other detection strategies and the corresponding results were listed in Table S2. This proposed assay obviously offers very high sensitivity due to the CPA-Cas 12a assisted double-amplification strategy.

Good specificity, which directly affects the clinical application prospect, is one of the indispensable characteristics to evaluate biosensor. Several other gram-negative, gram-positive, and candida bacteria were involved in the selectivity study of this proposed method. As illustrated in Table 1, benefiting from the specificity design of the two pairs of primers, this assay had good selectivity for the detection of *S. aureus*.

Overall, the above results proved that our lateral flow strip had high sensitivity and good selectivity for pathogen detection.

**Table 1:** Specificity evaluation of CPA and CPA-Cas 12a sensing strategy

Species	CPA	CPA-Cas 12a
MRSA	*MecA*(+) *nuc* (+)	*MecA*(+) *nuc* (+)
*S. aureus*	*MecA*(-) *nuc* (+)	*MecA*(-) *nuc* (+)
*S. pneumoniae*	-	-
*E. coli*	-	-
*P. aeruginosa*	-	-
*Candidaalbicans*	-	-
*E. faecalis*	-	-
*E. cloacae*	-	-
*Klebsiella oxytoca*	-	-
Mixture (*S. aureus+*)	*MecA*(-) nuc (+)	*MecA*(-) nuc (+)
Mixture (*S. aureus-*)	-	-
The First Chongqing EQC in 2020, No. 4	*MecA*(+) nuc (+)	*MecA*(+) nuc (+)
The Second National EQC in 2018, No. 3	*MecA*(+) nuc (+)	*MecA*(+) nuc (+)

**Note: Mixture (*S. aureus*+) represents the mixture suspension of the above bacteria containing*S. aureus***; **Mixture (*S. aureus*-) represents a mixture suspension of the above bacteria that do not contain*S. aureus***. **“+” indicates a positive test result. “-” indicates a negative result. EQC, external quality control**

### S. aureus analysis in clinical sample

To evaluate the clinical application potential of this CPA and Cas 12a mediated lateral flow assay, the No. 4 quality control (QC) sample of the first Chongqing external quality control (EQC) collected in 2020 and No. 3 QC sample of the second national EQC collected in 2018 were then assessed by the proposed lateral flow assay. The results (Table 1) indicated that all samples were distinguished as MRSA, which matched with results published online. Subsequently, 202 pieces of secretions collected from 166 infected patients were tested by the proposed lateral flow assay again and culture identification of these secretions was carried out simultaneously for comparison. The characteristics of these patients and their secretions are shown in Table S3. (1) The median age of 166 patients was 48 years old (range, 9 ~ 81 years), and 59.6% of them were males; (2) 39.2% of patients came from orthopedics and trauma centers and 39.2% of them came from the center of otorhinolaryngology respectively; (3) According to the diagnosis, 80.1% of the secretions derived from open wounds, and the rest of them originated from closed wounds; (4) According to culture identification, the positive rate of clinical samples was 13.4%, of which 12 samples contained gram-positive bacteria, 11 were *S. aureus* (2 were MRSA), and 1 sample contained *streptococcus*.

The result of 202 samples analyzed by CPA and CAP-Cas 12a mediated lateral flow assay are illustrated in Table 2. Compared with the traditional culture identification method, the CPA and CPA-Cas 12 A mediated lateral flow assays had a higher checkout rates of 6.4% (13 samples were identified by RT-PCR showed in Fig. S3), and the reasons were summarized as follows: (1) CPA and CPA-Cas 12a assay adopted molecular diagnostic have high sensitivity by extracting characteristic genes from *S. aureus* and amplifying corresponding DNA; (2) Traditional culture identification adopted smear methods that can only “feel” surface of a sample, so the inoculation is incomplete and there is a certain degree of false-negatives; (3) Compared with smear inoculation method, we adopt homogeneous reaction system, so the bacteria on secretion swabs was released completely; (4) CPA and CPA-Cas 12a based lateral flow biosensors employing isothermal amplification strategy could significantly amplify the signal of bacterial target gene and then enhance the sensitivity of bacteria detection.

According to the propose of original design, CPA-Cas 12a mediated lateral flow assay employing double amplification strategy should have higher sensitivity and lower detection limit than CPA mediated lateral flow assay that only amplifies signal one time theoretically. However, in our study, the checkout rate of CPA-Cas 12a based lateral flow assay for clinical samples was not significantly improved compared to that of CPA method, which is not an expected result. The possible reason may be attributed to this it that the infective doses of pathogenic bacteria in real samples is usually ranging from 10^2^ to 10^6^ CFU mL^− 1^ [27, 28]. This much higher quantity of bacteria than the detection limit of CPA-Cas 12a based method does not fully exert the advantage of high sensitivity of this method, which leaves us the impression here that the detection performance of both CPA-Cas 12a and CPA based lateral flow biosensors was comparable. Although the advantage of CPA-Cas 12a based lateral flow biosensor was not completely showed here, its ability for clinical testing had been demonstrated successfully.

**Table 2:** Test results of actual samples

	Culture identification	CPA	CPA-Cas 12a
The detection rate of *S. aureus*	11(5.4%)	13(6.4%)	13(6.4%)
The detection rate of MRSA	2(1.0%)	3(1.5%)	3(1.5%)

### Performance of CPA assay accommodated in microfluidic devices

**Fig. 6:**
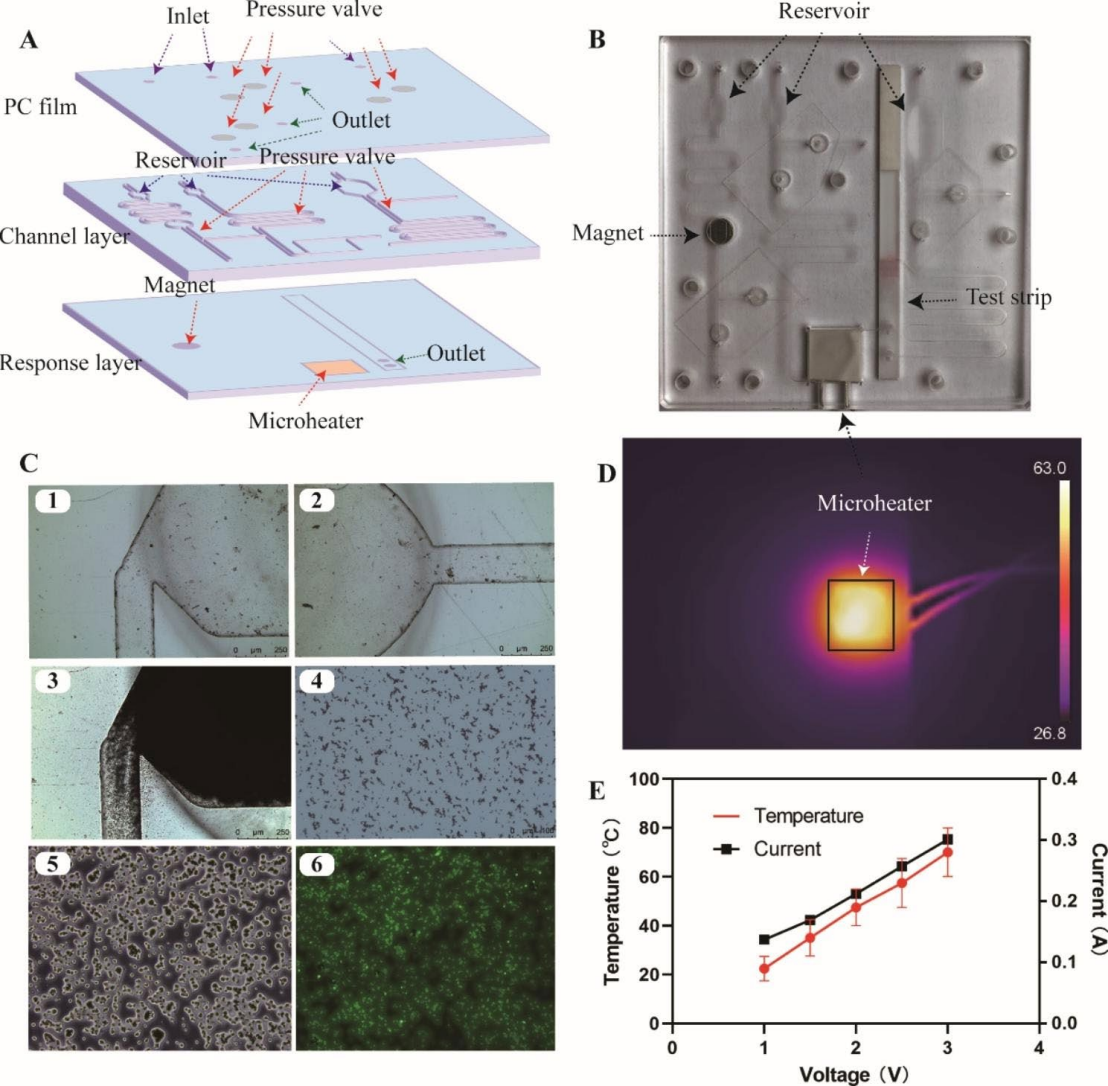
Schematic diagram and characterization of microfluidic device. **(A-B)** Structure diagram and digital image of microfluidic device. **(C)** Micrograph of magnetic bead enrichment cell and characterization of DNA isolation. **(D)** Microheater characterized by IR camera. **(E)** Current and temperature of the microheater powered at different voltages

A microfluidic device had been designed to integrate the CPA based sensing strategy to simplify the operation process of multiple genes detection of *S. aureus* in clinical samples and realize this process automatically. The schematic illustration of the microfluidic device is shown in Fig. 6A, and its corresponding digital image is shown in Fig. 6B. The structure of microfluidic device is composed of three layers, which are polycarbonate (PC) membrane layer, channel layer, and response layer respectively. Figure 6C1 and 6C2 are microscopic images of the magnetic bead enrichment cell. Figure 6C3 is the microscopic images of the magnetic bead enrichment cell filled with magnetic beads. The magnetic beads randomly distributed well in the enrichment cell as shown in Fig. 6C. The adsorption of FITC-labeled DNA on magnetic beads is shown in Fig. 6C5 and the corresponding green fluorescence signal derived from FITC could be obviously observed in Fig. 6C6. It proved that homodisperse magnetic beads had successfully adsorbed with DNA extracted from bacteria.

We then explored the voltage dependent temperature of microheater (R = 10 Ω) as shown in Fig. 6E. The increasing in the voltage correspondingly increased the current linearly, and then the temperature of microheater. Figure 6D shows a captured infrared image of the microdevice mounted on the system where the microheater driven by a DC power controller was used to heat the CPA chamber. When the voltage of the microheater was set at 2.1 V, the temperature of CPA reaction cell E was steadily maintained at about 63.0 °C at the central top area while the heat diffused to surrounding area and the temperature there was slightly lower than its counterpart. Then we injected clinical positive samples into this microfluidic device as above-mentioned for analysis. These samples were determined as 3 pieces of MRSA and 10 pieces of *S. aureus*, which agreed with previous detection results.

### Guiding clinical medication

*S. aureus* is able to access the underlying tissues or bloodstream to cause infection when the cutaneous and mucosal barriers are disrupted [16]. The antibiotics is usually employed to cure disease related to *S. aureus*. However, the widespread use of spectrum antibiotics might lead to an increase in drug resistance, and their use should be under monitor for drug resistance during treatment. Therefore, early microbiological diagnosis of *S. aureus* infections and detection of drug resistance are essential for the efficient treatment of disease.

To verify the usefulness of our proposed assay for the guideline of clinical antibiotics medication, the detection results of secretions collected from patients were given back to their clinicians as well as some evidence for rational use of antibiotics. The clinical data and antibiotic usage of 13 *S. aureus* infected patients (P_1_-P_13_) were collected and shown in Table S4. 10 (76.92%) of infected patients were empirically administered antibiotics upon receipt of analysis results. Most selected antibiotics were penicillin or second-generation cephalosporin antibiotics combined with mupirocin or fusidic acid. This treatment regimen was used to treat infections by most gram-negative bacteria and gram-positive bacteria. In particular, the topical antibiotics of mupirocin and fusidic acid were especially effective for skin or wound infections caused by gram-positive bacteria.

Due to poor physical condition and severe infection of P_1_ and P_9_ patients, clinicians chose cefuroxime or piperacillin-sulbactam combined with vancomycin for anti-infection therapy at the early stage of infection, which covered most gram-negative and positive bacteria, and eventually achieved good efficacy. This demonstrated that the evidence of *S. aureus* infection we provided within 3 h was very important for further confirming the clinician’s choice of antibiotics.

In addition, there was a case of inconsistency between analysis results and clinical symptoms. The P_11_ patient had no obvious sign of MRSA infection, while MRSA was detected from its nasopharyngeal secretion. It might be attributed to colonization bacteria or sample contamination. *S. aureus* is found in the human commensal microbiota of the nasal mucosa in 20–40% of the general population[32].

Moreover, debridement and regular wound care are essential for recovery of infected patient. Each patient had received effective debridement, cleaning or changing dressing of infected site, which could effectively inhibit the growth of pathogenic bacteria. Although the P_4_ patient did not use antibiotics, the regular clean of necrotic tissue and wound in the nasal cavity is useful for treatment. The plantar infected patient (P_8_) was given antifungal treatment by doctor, but our result indicated that he was infected by *S. aureus*. Although there is a contradiction, the P_8_ patient still recovered well due to carefully daily debridement without changing his treatment regimen. Unfortunately, homogenization analysis of antibiotics therapeutic effect could not be implemented because different patients have diverse statuses. In a word, these results imply that the proposed assay for MRSA and *S. aureus* has a promising feature for antibiotic selection for physicians within 3 h.

## Conclusion

A CPA-Cas 12a sensing strategy was developed for visual detection of *S. aureus* and MRSA with high sensitivity and selectivity. This method takes advantage of both CPA and CRISPR Cas 12a to effectively amplify target signal. The employment of CPA and CRISPR Cas 12a amplification strategies in the single lateral flow strip had resulted in an inexpensive and disposable paper-based device as a general point-of-care testing tool for bacteria detection. Moreover, the integration of paper-based strip with a microfluidic device to implement the on-chip extraction and enrichment of DNA from bacteria, isothermal nucleic acid amplification and detection further simplifies sample pretreatment and detection procedure. This proposed CPA-Cas 12a based lateral flow strip was capable of detecting *S. aureus* within 180 min with an LOD of 5 CFU mL^− 1^. It also showed a good agreement of detection for clinical sample with published quality control information, indicating the suitability of the method in clinical diagnostics. Furthermore, with the simple, rapid, and sensitive CPA-Cas 12a lateral flow assay, timely diagnostics of *S. aureus* in clinical setting could help physicians to prescribe clinical antibiotics. This work had overcome the drawbacks of poor sensitivity and high rates of false-negative results that most current colorimetric paper lateral flow assays suffered. By careful designing primer, the proposed CPA-Cas12a sensing strategy is expected to have great potential for portable, rapid, and sensitive analysis of many other bacteria in clinical diagnostics and antibiotics prescription.

## Electronic supplementary material

Below is the link to the electronic supplementary material.

Supplementary Material 1
