# Supplementary material for: CPA-Cas12a-based lateral flow strip for portable assay of Methicillin-resistant Staphylococcus aureus in clinical sample
Source: J Nanobiotechnology. 2023 Jul 22;21:234. doi: 10.1186/s12951-023-02002-1 (PMC10362775; doi:10.1186/s12951-023-02002-1)
Supplement: Supplementary file 1 — Supplementary Material 1 [file 12951_2023_2002_MOESM1_ESM.docx]

**Supplementary Material**

CPA-Cas12a-based lateral flow strip for portable assay of methicillin-resistant *Staphylococcus aureus* in clinical sample

Jiangling Wu ^a,#^, Yu Huang ^b,c,#^, Xiaojuan Ding ^d,#^, Lina Kang ^a^, Xiaoliang Wang ^a^, Dandan Li ^d^, Wei Cheng ^e,*^,Gang Liu ^f,*^,JianjiangXue ^a,*^, Shijia Ding ^g^

a Department of Clinical Laboratory, Medical Sciences Research Center, University-Town Hospital of Chongqing Medical University, Chongqing, 401331, China

b Chongqing Key Laboratory of Multi-scale Manufacturing Technology, Chongqing Institute of Green and Intelligent Technology, Chinese Academy of Sciences, Chongqing, 400714, China

c Chongqing School, University of Chinese Academy of Science, Chongqing, 400714, China

d Department of Clinical Laboratory, The Second Affiliated Hospital of Chongqing Medical University, Chongqing, 401331, China

e The Center for Clinical Molecular Medical Detection, The First Affiliated Hospital of Chongqing Medical University, Chongqing,400016, China

f Department of Critical Care Medicine, University-Town Hospital of Chongqing Medical University, Chongqing, 401331, China

g Key Laboratory of Clinical Laboratory Diagnostics (Ministry of Education), college of laboratory medicine, Chongqing Medical University, Chongqing,400016, China

# These authors contributed equally to this work

*Corresponding author email address: chengwei@hospital.cqmu.edu.cn (W. Cheng), lg@hospital.cqmu.edu.cn (G. Liu) and jianjiangxue@126.com (J. Xue)

**Table of Contents**

Table S1. Sequences of DNA oligonucleotides used in this work……………………3

Table S2. Comparison of CPA-Cas 12a assay for the detection of *S. aureus*………...4

Table S3. Basic characteristics of patients……………………………………………5

Table S4. Antibiotic usage in 13 patients with S. aureus infection………………..….6

Fig. S1. Schematic diagram and electrophoretic verification of CPA single double signal amplification system ………....……………………………………………………...7

Fig. S2. Agarose gel of double-crossing CPA combined with CRISPR-Cas 12a….....8

Fig. S3. Standard real-time quantitative PCR with 13 samples…………………..…9

**Table S1. Sequences of DNA oligonucleotides used in this work.**

| **Nucleic acid sequence** | **sequence (5'-3')** |
| --- | --- |
| mecA-CF | AGGTTCTTTTTTATCTTCGGTTA-CAACATGAAAAATGATTATGGCTC (CD2-CD1) |
| mecA-CB | CAACATGAAAAATGATTATGGCTC-AGGTTCTTTTTTATCTTCGGTTA  (CD1-CD2) |
| FITC-DF | FITC-TGCTAAAGTTCAAAAGAGTATTTAT (DF) |
| Biotin-DB | Biotin-AGTTGTAATCTGGAACTTGTTGAGC (DB) |
| mecA-DF | TGCTAAAGTTCAAAAGAGTATTTAT (DF) |
| mecA-DB | AGTTGTAATCTGGAACTTGTTGAGC (DB) |
| nuc-CF | AGTTCTTTGACCTTTGTCAAACTCG-GTCAACCAATGACATTCAGACTAT (CD2-CD1) |
| nuc-CB | GTCAACCAATGACATTCAGACTAT-AGTTCTTTGACCTTTGTCAAACTCG (CD1-CD2) |
| Dig-DF_2_ | Dig-GTGATACGGTTAAATTAATGTACAA (DF) |
| Biotin-DB_2_ | Biotin-AGTTGTAATCTGGAACTTGTTGAGC (DB) |
| nuc-DF_2_ | GTGATACGGTTAAATTAATGTACAA (DF) |
| nuc-DB_2_ | AGTTGTAATCTGGAACTTGTTGAGC (DB) |
| mecA-CrRNA | *UAAUUUCUACUAAGUGUAGAU*UGUAUGGCAUGAGUAACGAAG |
| nuc-CrRNA | *UAAUUUCUACUAAGUGUAGAU*CGAAAAAAAUGGUAGAAAAUG |
| Beacon | Fam-TTATTATTATT-Biotin |
| Beacon_2_ | Fam-TTATT-Dabcyl |

**Note: CrRNA consists of two parts, a fixed sequence (Italic) and a variable region (Underline).**

**Table S2. Comparison of CPA-Cas 12a assay for the detection of *S. aureus*.**

| **Biosensing strategies** | **LOD （CFU mL^-1^）** | **Time (min)** | **Ref.** |
| --- | --- | --- | --- |
| Magnetic nanobeads and gold  surface based paper sandwich-type biosensor | 2 | 120 | [1] |
| label-free aptamer based LSPR biosensor | 1000 | 2 | [2] |
| RT-PCR | 1000 | 120 | [3] |
| Aptamer based sandwich-type electrochemical biosensor | 39 | several | [4] |
| aptamer and vancomycin-copper nanoclusters based fluorescence biosensor | 80 | 35 | [5] |
| Upconversion nanoprobes based horseradish peroxidase-regulated dual-mode fluorescence biosensor | 22 | several | [6] |
| CRISPR/Cas12a based fluorescence-enhanced lateral flow biosensor | 540 | 70 | [7] |
| CRISPR Cas12a based lateral flow | 10 | 70 | [8] |
| CPA-Cas 12a based biosensor | 5 | 180 | This work |

**Note: LSPR, Localized surface plasmon resonance sensing; RT-PCR, Real-time polymerase chain reaction**

**Table S3. Basic characteristics of patients（n=166）**

| **Characteristics** | **n（%）** |
| --- | --- |
| Median age (range) | 48（9-81） |
| Male | 99（59.6） |
| Female | 67（40.4） |
| Orthopedics and Trauma Center | 65（39.2） |
| Otolaryngology center | 65（39.2） |
| Open wound | 133（80.1） |
| Closed wound | 33（19.9） |
| Positive rate | 27（13.4） |
| G^+^ bacterial infection | 12（6.0） |
| Checkout rate of *S. aureus* | 11（5.4） |
| The detection rate of MRSA | 2（1.0） |

**Table S4. Antibiotic usage in 13 patients with *S. aureus* infection**

| No. | Name | Gender | Age | Ward | Name of result | Antibiotic selection | Testing result |
| --- | --- | --- | --- | --- | --- | --- | --- |
| 1 | Li** | Female | 60 | Nerve center | Scalp abscess (left frontal top) | Cefuroxime &Vancomycin | *S. aureus* |
| 2 | Gun** | Male | 69 | Nephropathy urinary center | Subcutaneous abscess | Piperacillin-sulbactam &Mupirocin | *S. aureus* |
| 3 | Huang** | Female | 26 | Otorhinolaryngologic Department | Rhinitis | Cefuroxime | *S. aureus* |
| 4 | Li** | Male | 20 | Otorhinolaryngologic Department | Chronic sinusitis | Unused | *S. aureus* |
| 5 | Shi** | Male | 32 | Otorhinolaryngologic Department | External auditory canal mass | Cefuroxime | *S. aureus* |
| 6 | Fan** | Male | 47 | Digestive center | An abscess in the groin | Cefuroxime & Fusidic acid | *S. aureus* |
| 7 | Ren** | Male | 30 | Digestive center | Postoperative wound infection | Mupirocin | *S. aureus* |
| 8 | Dai** | Female | 26 | Bone and Trauma Center | Foot skin infection | Antifungal agent | *S. aureus* |
| 9 | Xia* | Male | 25 | Bone and Trauma Center | Multiple fractures with infection | Piperacillin-sulbactam &Vancomycin; DEAT：Amoxicillin-clavulanate potassium | *S. aureus* |
| 10 | Zheng* | Male | 60 | Bone and Trauma Center | Infection of finger | Cefuroxime | *S. aureus* |
| 11 | Zhang** | Female | 59 | Otorhinolaryngologic Department | Nosebleed | Unused | MRSA |
| 12 | Hu** | Male | 63 | Bone and Trauma Center | Soft tissue infection of foot | Penicillin G& Amoxicillin-clavulanate potassium | MRSA |
| 13 | Yang** | Male | 36 | Bone and Trauma Center | Wound infection | Cefuroxime & Fusidic acid | MRSA |

**
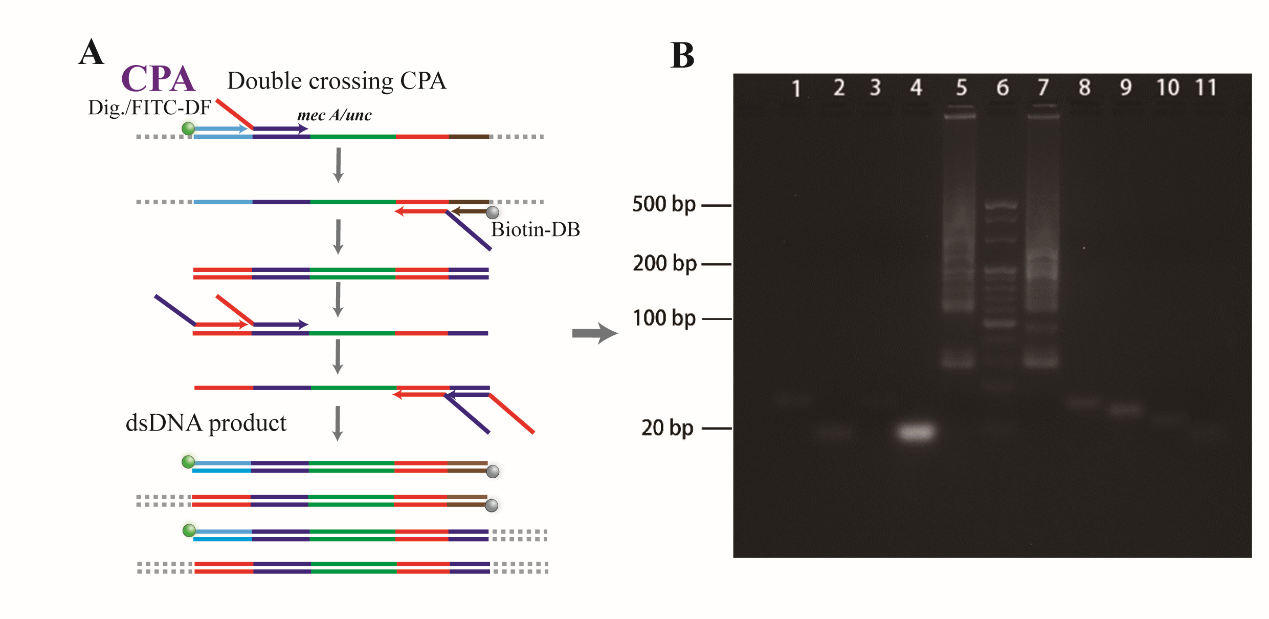
**

**Fig. S1. (A) Schematic diagram and (B) electrophoretic verification of CPA single double signal amplification system.**


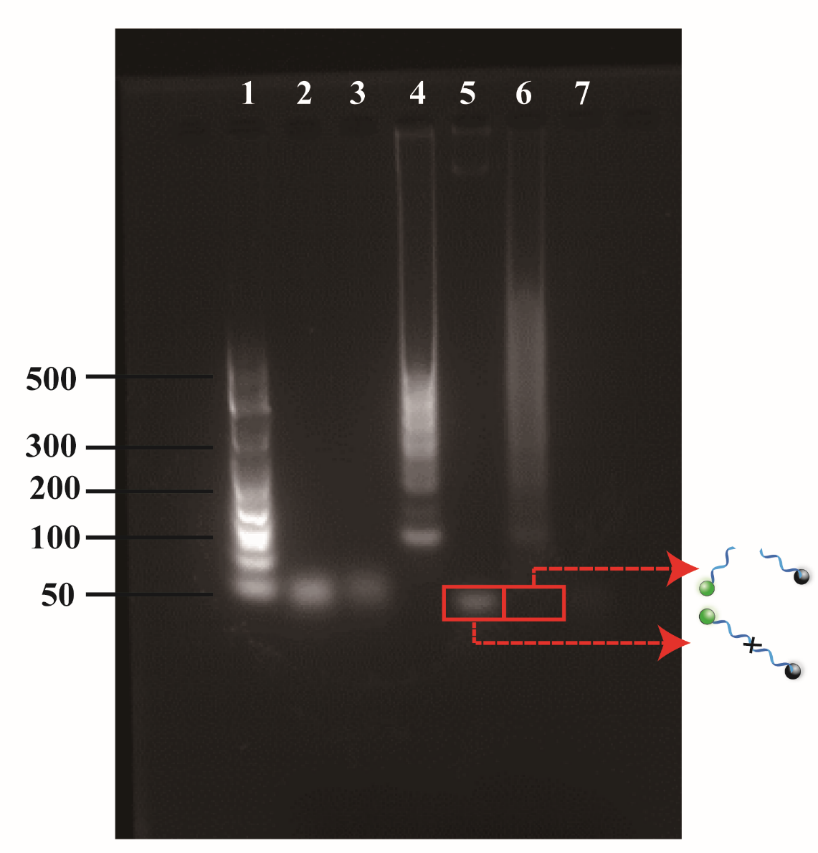


**Fig. S2. Agarose gel of double-crossing CPA combined with CRISPR-Cas 12a. Lane 1 was marker, lane 2 was CrRNA, lane 3 was CrRNA+Cas12a, lane 4 was CPA product, lane 5 was DNA reporter molecule, and lane 6 was CPA-Cas 12a double amplification system reaction product, lane 7 was the negative control without CPA product.**


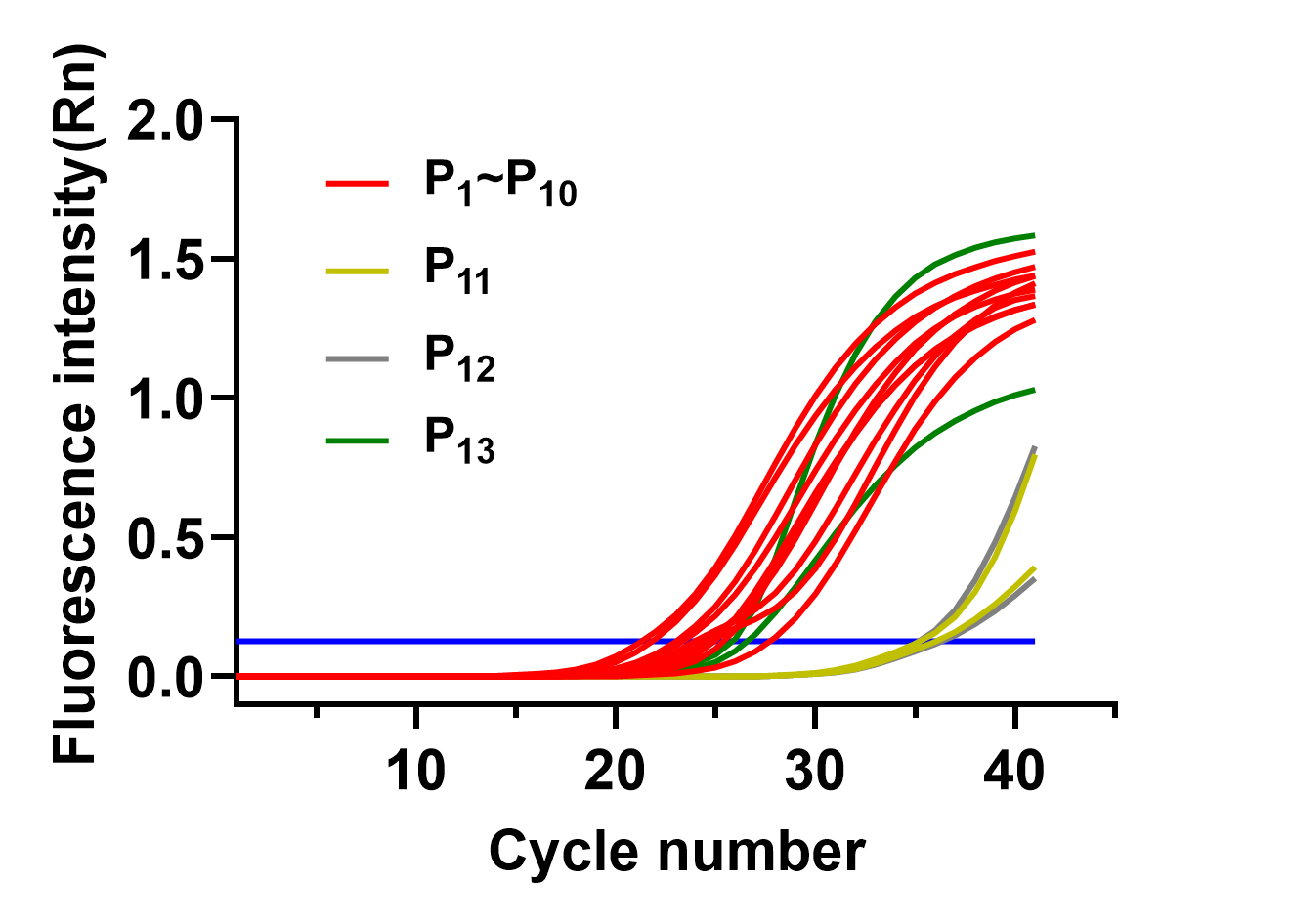


**Fig. S3. Standard real-time quantitative PCR with 13 samples**

**Reference:**

1. Suaifan GARY, Alhogail S, Zourob M. Rapid and low-cost biosensor for the detection of Staphylococcus aureus. Biosens Bioelectron. 2017;90:230–7.

2. Khateb H, Klös G, Meyer RL, Sutherland DS. Development of a Label-Free LSPR-Apta Sensor for Staphylococcus aureus Detection. ACS Appl Bio Mater. 2020;3:3066–77.

3. Alarcón B, Vicedo B, Aznar R. PCR-based procedures for detection and quantification of Staphylococcus aureus and their application in food. J Appl Microbiol. 2006;100:352–64.

4. Nguyen TT-Q, Kim ER, Gu MB. A new cognate aptamer pair-based sandwich-type electrochemical biosensor for sensitive detection of Staphylococcus aureus. Biosens Bioelectron. 2022;198:113835.

5. Bagheri Pebdeni A, Mousavizadegan M, Hosseini M. Sensitive detection of S. Aureus using aptamer- and vancomycin -copper nanoclusters as dual recognition strategy. Food Chem. 2021;361:130137.

6. Ouyang Q, Wang L, Ahmad W, Yang Y, Chen Q. Upconversion Nanoprobes Based on a Horseradish Peroxidase-Regulated Dual-Mode Strategy for the Ultrasensitive Detection of Staphylococcus aureus in Meat. J Agric Food Chem. 2021;69:9947–56.

7. Zhou B, Ye Q, Li F, Xiang X, Shang Y, Wang C, et al. CRISPR/Cas12a based fluorescence-enhanced lateral flow biosensor for detection of Staphylococcus aureus. Sens Actuators B Chem. 2022;351:130906.

8. Qian J, Huang D, Ni D, Zhao J, Shi Z, Fang M, et al. A portable CRISPR Cas12a based lateral flow platform for sensitive detection of Staphylococcus aureus with double insurance. Food Control. 2022;132:108485.
